# Nasolabial and distal limbs dry gangrene in newborn due to hypernatremic dehydration with disseminated intravascular coagulation: a case report

**DOI:** 10.1186/s40748-022-00140-2

**Published:** 2022-08-29

**Authors:** Ayanaw Tamene, Yalemwork Anteneh, Haimanot Amare, Yihunie Yerdaw

**Affiliations:** 1 Department of Pediatrics and Child Health, College of Medicine and Health Sciences, Bahir Dar University, Bahir Dar, Ethiopia; 2 Department of Pediatrics and Child Health, Debre Tabor University, Bahir Dar, Ethiopia

**Keywords:** Gangrene, Dehydration, Hypernatremia, Neonate

## Abstract

**Introduction:**

Gangrene is the death of an organ or tissue due to lack of blood supply or bacterial infection. In neonates, gangrene is usually caused by sepsis, dehydration, maternal diabetes, asphyxia, or congenital anticoagulant deficiency. It commonly occurs in the extremities. Gangrene may lead to death or amputation of the limb. Early diagnosis and prompt management of the underlying cause halts the progression of the disease.

**Case presentation:**

A 12-day-old neonate presented with a complaint of black discoloration of the nose and feet for 2 days. He was breastfeeding poorly and had signs of dehydration. Upon physical examination, he was tachycardic (pulse rate = 182 beats per minute), tachypneic (respiratory rate = 62 breaths per minute), and hypothermic (temperature = 35.0 oC). He lost 33.3% of his birth weight. He had demarcated cold, dry, and dark discoloration of the entire nose, nasal septum; upper lip; palate; bilateral distal lower limbs; and the left fifth finger. Dorsalis pedis arteries were not palpable on either side. On investigation, the baby had pancytopenia, hypernatremia, elevated creatinine, elevated coagulation profiles, and absent arterial flow in bilateral dorsal pedis arteries. He was treated for hypernatremic dehydration and possible sepsis. He was transfused with whole blood, platelets, and fresh frozen plasma, but finally, the patient passed away on the 7th day of admission.

**Conclusion:**

The entire nose, upper lip, soft and hard palate, symmetric lower limb, and fifth finger gangrene due to severe hypernatremic dehydration complicated by disseminated intravascular coagulation may occur in the same patient. To avoid such serious neonatal problems, mothers should be properly educated about optimal breastfeeding techniques and schedule well-child visits 3–5 days after birth.

## Introduction

Gangrene is the death or decay of an organ or tissue due to lack of blood supply or serious bacterial infection [1, 2]. Gangrene can be dry or wet. Dry gangrene is due to a sudden loss of arterial supply to a tissue or organ. It involves dry and shriveled skin that looks brown to purplish-blue or black. Wet gangrene is caused by a bacterial infection in the affected tissue which manifests with swelling, blistering, and a wet appearance [3]. Gangrene in neonates occurs rarely, but it has serious consequences [4–6]. Common causes of gangrene in neonates are sepsis, dehydration, maternal diabetes, indwelling arterial or venous catheter, vasculitis, hypernatremic dehydration, polycythemia, syphilis, cold exposure, hyperglycemia, asphyxia, intravenous hyperosmolar infusion, hereditary thrombotic disorders, antiphospholipid antibody syndrome, congenital heart defect, and neonatal diabetes mellitus [7–11]. However, in the majority of cases, an etiological factor is not identified [2, 6, 12].

Neonatal gangrene usually occurs over the extremities, which can be unilateral or bilateral, symmetric or asymmetric [1, 7, 13, 14]. Gangrene over the umbilicus, buttock, nose, and lip can occur in rare cases during neonatal age [4, 5, 15]. Gangrene usually occurs after birth, but it might also occur in the intrauterine environment [8]. Gangrene generally has poor outcomes, leading to death or loss of a limb [3]. Early diagnosis and prompt management of the underlying cause can halt the disease progression [14].

We present a rare case report of gangrene of the entire nose, upper lip, and palate, bilateral symmetric lower limb, and left fifth finger due to hypernatremic dehydration with disseminated intravascular coagulation.

Case presentation: A 12-day-old male neonate who was born from a 26-year-old para I mother at a gestation age of 40 + ^1^ weeks was referred from the primary hospital with a complaint of black discoloration of the nose and feet of 2 days’ duration. The mother had antenatal care at a local health center, and the pregnancy was uneventful. She was negative for VDRL, HBSAg, and HIV tests during pregnancy. The labor started spontaneously. The mode of delivery was spontaneous vertex delivery after 10 hours of labor and 4 hours of rupture of the membrane, respectively. The birth outcome was a 3000 g male neonate with an APGAR score of 7 and 9 at the 1st and 5th minute, respectively. The baby started breastfeeding after 1 h and was discharged 6 h later. He was breastfeeding poorly. On the 10th day of life, the mother noticed a black discoloration at the tip of the nose. On the next day, the black discoloration on the nose increased in size and involved the upper lip. Associated with this was also a history of low-grade fever, fast breathing, grunting, excessive crying, and decreased breastfeeding. Otherwise, he had no history of abnormal body movement or loss of consciousness. For the above illness, they visited the nearby primary hospital and were given 2 doses of ampicillin and gentamycin intravenously and were referred to our hospital.

**Fig. 1:**
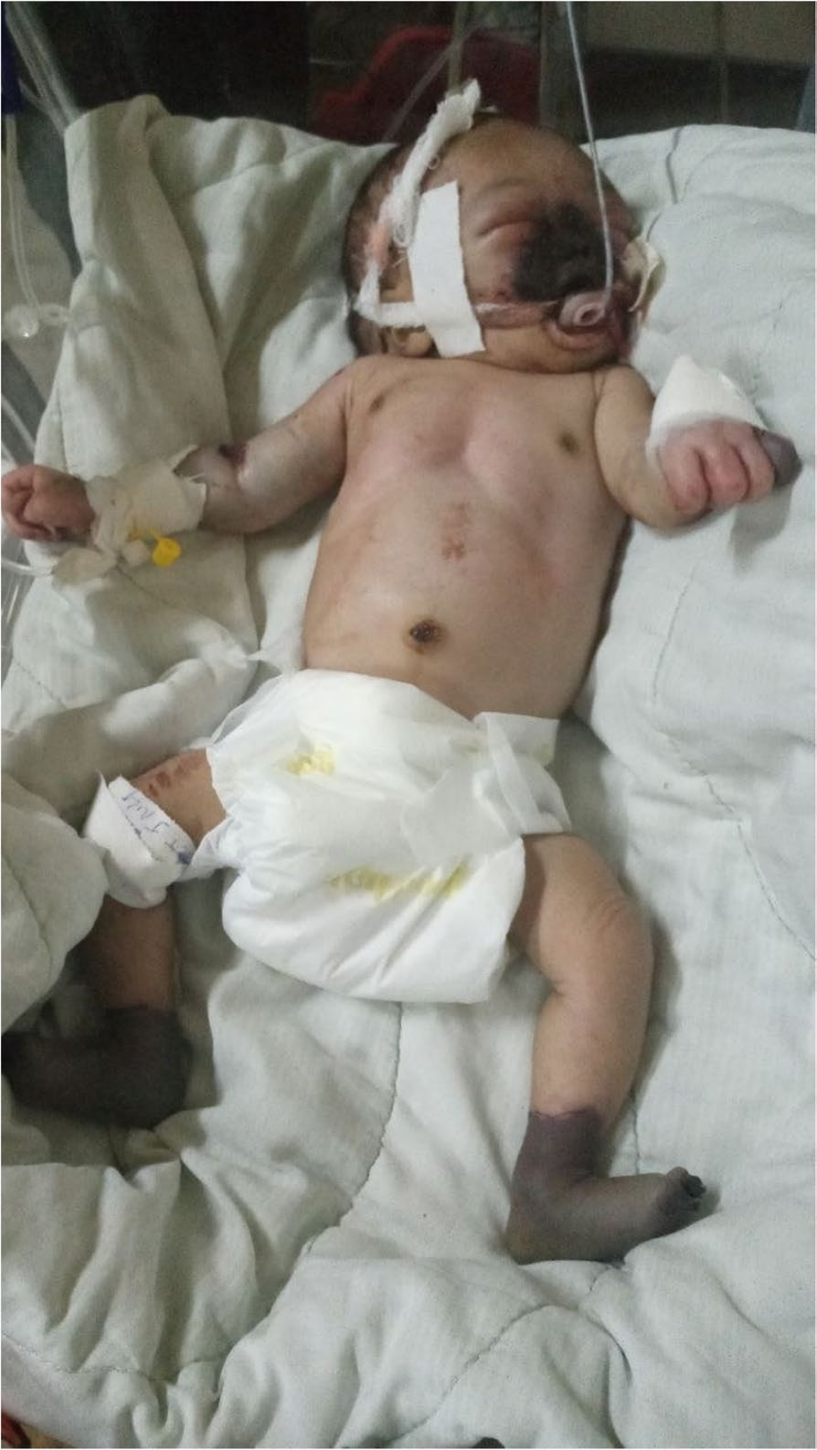
Shows dry darkish discoloration of the entire nose, upper lip, bilateral feet, and distal legs, and left the fifth finger. There is also a bluish discoloration and swelling of the right elbow joint due to subcutaneous hematoma after removal of intravenous cannula site (Mobile phone picture taken on 5th day of admission)

On arrival at our hospital, pulse rate = 182 beats per minute (tachycardia), respiratory rate was 62 breaths per minute (tachypneic), T^0^ = 35 °C (hypothermic), weight = 2000 g (33.3% loss of birth weight). The oxygen saturation was below 90% on room air. He had a depressed fontanel, sunken eyeballs, and dry lips. He had demarcated cold, dry, and dark discoloration of the entire nose, nasal septum, upper lip; and hard and soft palate. On the musculoskeletal examination, he had cold and dark discoloration of the feet and distal legs up to 4 cm proximal to the medial malleoli bilaterally. In addition, the left fifth finger was dark up to the level of the metacarpophalangeal joint, as shown in Fig. 1. Dorsalis pedis arteries were not palpable on either side. There was no blistering or crepitation over the dark areas. The skin was doughy with a very slow skin pinch. He was lethargic with depressed primitive neonatal reflexes. The reminder of physical examinations was normal. Random blood glucose was 380 mg/dl at arrival at the hospital. On complete blood count, he initially had neutrophilia and later progressive pancytopenia. The coagulation profiles were deranged. As a result of the serum electrolyte test, there was severe hypernatremia (Table 1). The urine output was 0.8 ml/kg/hour at admission, but later he became anuric. The serum creatinine was 2.99 mg/dl (the normal value for age is 0.03–0.4 mg/dl). In the Doppler ultrasound study, there was no arterial flow in the bilateral dorsal pedis arteries. Otherwise, there was spectral internal color flow over other arteries. Transfontanel sonography was normal. Due to a lack of resources, urine sodium, fractional excretion of sodium, arterial blood gases, and D-dimer were not done.

**Table 1:** Serial complete blood cell count and serum electrolyte result of the patient

Investigation		Days of admission					Normal reference value
		Day 1 04/11/2021	Day 2 05/11/2021	Day 4 07/11/2021	Day 6 09/11/2021	Day 7 10/11/2021	
Complete blood count	White blood cell count	23.66 × 10^3^	33.2 × 10^3^	4.53 × 10^3^	4.41 × 10^3^	2.93 × 10^3^	9.1–34 × 10^3^
	Absolute neutrophil count	18.88 × 10^3^	23.67 × 10^3^	3.42 × 10^3^	3.53 × 10^3^	2.09 × 10^3^	3.5–7.5 × 10^3^
	Hematocrit	57.7%	46.6%	36.5%	36.1%	26.5%	45–69%
	Platelet count	84 × 10^3^	72 × 10^3^	7 × 10^3^	18 × 10^3^	3 × 10^3^	150–450 × 10^3^
Serum electrolyte	Sodium (Na+)	191 mmol/L	179 mmol/L	156 mmol/L	152 mmol/L	157 mmol/L	135–145 mmol/L
Coagulation profile	PT		15.1 seconds		21.3		10–15 seconds
	APTT		64.3 seconds		100.2		23–38 seconds
	INR		1.59		3.6		0.94–1.3
	Fibrinogen		92 mg/dl		83 mg/dl		

He was put on intranasal oxygen, ampicillin, and ceftriaxone, which was later changed to ceftriaxone, vancomycin (adjusted dose for creatinine clearance), and metronidazole. He was on maintenance fluid and kept NPO due to hypoactive bowel sound. On the first day of admission, he developed coffee ground gastric content, dark stool, and prolonged bleeding from needle prick sites despite therapeutic vitamin K administration. The baby was treated for hypernatremic dehydration via intravenous fluid. He was transfused with 20 ml/kg of whole blood and then 02 units of platelets and one unit of fresh frozen plasma. The hyperglycemia was treated with regular insulin at 0.05–0.1 IU/kg/hour and it was corrected after 24 hours of treatment. The regular insulin was discontinued. The dark discoloration of the palate progressed to the soft palate and tonsillar areas, resulting in worsening respiratory distress. The intranasal oxygen was changed to face and 8 cmH2O bubble CPAP as the distress worsened progressively. Despite the above management, the patient’s condition deteriorated and passed away on the 7th day of admission.

## Discussion

Gangrene is the death or decay of an organ or tissue due to lack of blood supply or serious bacterial infection [2]. Gangrene can be dry or wet. Dry gangrene is due to a sudden loss of arterial supply to a tissue or organ. It involves dry and shriveled skin that looks brown to purplish-blue or black. In our case, the gangrenous areas were dry and had no blistering or crepitation. Even though gangrene is rare in neonates, it is one of the peak ages for gangrene in pediatrics. Neonatal gangrene has serious consequences [3–6]. Neonatal gangrene usually occurs over the extremities, which can be unilateral or bilateral, symmetric or asymmetric [1, 7, 13, 14]. In our case, the neonate had symmetric lower limb gangrene and asymmetric fifth digit upper limb gangrene. Gangrene has generally poor outcomes, leading to death or limb loss [3]. In our case, the nasolabial gangrene progressed and led to worsening respiratory distress and finally to death.

Common causes of gangrene in neonates are sepsis, dehydration, maternal diabetes, indwelling arterial or venous catheter, vasculitis, hypernatremic dehydration, polycythemia, syphilis, cold exposure, hyperglycemia, asphyxia, intravenous hyperosmolar infusion, hereditary thrombotic disorders, antiphospholipid antibody syndrome, congenital heart defect, and neonatal diabetes mellitus [6–12]. Our patient had severe hypernatremic dehydration secondary to inadequate feeding. He lost 33.3% of his birth weight within 12 days. Being primiparous and a lack of training on proper positioning and attachment by health professionals could lead to this suboptimal breastfeeding and its complications. Showing the proper positioning and attachment and counseling about signs of optimal breastfeeding should be practiced for all mothers in general and primiparous mothers in particular, in a setup where there is no routine newborn reevaluation after discharge. The patient had acute kidney injury, likely due to dehydration and or disseminated intravascular coagulation.

The management of gangrene in neonates usually includes treating the underlying etiology to halt the disease progression, intravenous antibiotics, fresh frozen plasma, heparin based anticoagulation, the substitution of natural anticoagulants, and amputation of the gangrened limb. Early recognition, prompt management of DIC, and treating the underlying conditions may halt the progression of the disease [2, 15, 16]. Our patient had hyperglycemia, which was corrected after treatment, which was likely due to severe hypernatremia and the stressful condition of the patient [10, 17, 18].

## Conclusion

We present a rare case report of dry gangrene of the entire nose, upper lip, soft and hard palate, symmetric distal lower limbs, and fifth finger due to hypernatremic dehydration complicated by disseminated intravascular coagulation in the same patient. To avoid such serious neonatal problems, mothers should be properly educated about optimal breastfeeding techniques and schedule well-child visits 3–5 days after birth.

## Data Availability

The datasets used in this article are available from the corresponding author on reasonable request.
